# Bats, Trypanosomes, and Triatomines in Ecuador: New Insights into the Diversity, Transmission, and Origins of *Trypanosoma cruzi* and Chagas Disease

**DOI:** 10.1371/journal.pone.0139999

**Published:** 2015-10-14

**Authors:** C. Miguel Pinto, Sofía Ocaña-Mayorga, Elicio E. Tapia, Simón E. Lobos, Alejandra P. Zurita, Fernanda Aguirre-Villacís, Amber MacDonald, Anita G. Villacís, Luciana Lima, Marta M. G. Teixeira, Mario J. Grijalva, Susan L. Perkins

**Affiliations:** 1 Centro de Investigación en Enfermedades Infecciosas, Pontificia Universidad Católica del Ecuador, Quito, Ecuador; 2 Sackler Institute for Comparative Genomics, American Museum of Natural History, New York, New York, United States of America; 3 Department of Mammalogy, American Museum of Natural History, New York, New York, United States of America; 4 The Graduate Center, The City University of New York, New York, New York, United States of America; 5 Division of Mammals, National Museum of Natural History, Smithsonian Institution, Washington, District of Columbia, United States of America; 6 Tropical Disease Institute, Department of Biomedical Sciences, Heritage College of Osteopathic Medicine, Ohio University, Athens, Ohio, United States of America; 7 Fundación Otonga, Quito, Ecuador; 8 Departamento de Parasitologia, Instituto de Ciencias Biomédicas, Universidade de São Paulo, São Paulo, São Paulo, Brazil; Tulane University, UNITED STATES

## Abstract

The generalist parasite *Trypanosoma cruzi* has two phylogenetic lineages associated almost exclusively with bats—*Trypanosoma cruzi* Tcbat and the subspecies *T*. *c*. *marinkellei*. We present new information on the genetic variation, geographic distribution, host associations, and potential vectors of these lineages. We conducted field surveys of bats and triatomines in southern Ecuador, a country endemic for Chagas disease, and screened for trypanosomes by microscopy and PCR. We identified parasites at species and genotype levels through phylogenetic approaches based on 18S ribosomal RNA (18S rRNA) and cytochrome b (cytb) genes and conducted a comparison of nucleotide diversity of the cytb gene. We document for the first time *T*. *cruzi* Tcbat and *T*. *c*. *marinkellei* in Ecuador, expanding their distribution in South America to the western side of the Andes. In addition, we found the triatomines *Cavernicola pilosa* and *Triatoma dispar* sharing shelters with bats. The comparisons of nucleotide diversity revealed a higher diversity for *T*. *c*. *marinkellei* than any of the *T*. *c*. *cruzi* genotypes associated with Chagas disease. Findings from this study increased both the number of host species and known geographical ranges of both parasites and suggest potential vectors for these two trypanosomes associated with bats in rural areas of southern Ecuador. The higher nucleotide diversity of *T*. *c*. *marinkellei* supports a long evolutionary relationship between *T*. *cruzi* and bats, implying that bats are the original hosts of this important parasite.

## Introduction

The protozoan parasite *Trypanosoma cruzi* (order Kinetoplastida) causes Chagas disease, one of the main tropical diseases in the Americas [1]. The intraspecific genetic variation of this species is complex, and several attempts have been made to establish main intraspecific lineages. Currently, *T*. *cruzi* is divided in two subspecies: *T*. *c*. *cruzi* and *T*. *c*. *marinkellei* [2], and even further, *T*. *c*. *cruzi* is divided in seven discrete typing units (DTUs) (i.e., TcI-TcVI and Tcbat) [3–6]. Moreover, new investigations have unveiled pronounced structuring within some DTUs e.g., [7–10]. However, among these subdivisions of *T*. *cruzi*, those lineages associated almost exclusively with bats (i.e., Tcbat and *T*. *c*. *marinkellei*) are arguably the most enigmatic for their biology and scant documentation.

*T*. *c*. *marinkellei* is restricted to bats, does not infect laboratory mice, and is thought to be exclusively transmitted by triatomines of the genus *Cavernicola* [11,12]. Nevertheless, the high prevalence and wide distribution of this trypanosome subspecies suggest the participation of other vectors [13]. In contrast, Tcbat like all *T*. *c*. *cruzi* DTUs, proved to be infective to mice [3]. In addition, Tcbat was recently found in a Colombian child and in pre-Columbian mummies of the Cabuza and Camarones cultures in Chile [14,15]. Tcbat is unable to develop in *T*. *infestans* and *R*. *prolixus* [3], and although its vectors remain to be discovered, the occurrence of this genotype in Brazil, Panamá, and Colombia suggests that it can be transmitted by diverse vector species.

Phylogenetic studies have demonstrated that Tcbat is sister to TcI, and it has been long established that *T*. *c*. *marinkellei* is sister to the monophyletic group formed for all DTUs [3,5,6,16]. Given that *T*. *cruzi* sensu lato belongs to the *T*. *cruzi* clade, a group of ~18 species that mostly parasitize bats [17–19], here we hypothesized that bats are the ancestral hosts of *T*. *cruzi*. Thus, it would be expected that the bat lineages of *T*. *cruzi*—specially the most basal lineage that is *T*. *c*. *marinkellei*—would have a greater genetic diversity than the other subdivisions of *T*. *cruzi*.

Here we provide information on the genetic variation, geographic distribution, host associations, and potential vectors of bat lineages of *T*. *cruzi*. We analyze the nucleotide diversity of the *T*. *cruzi* lineages to test the hypothesis that bats are the ancestral hosts of this parasite, and discuss the implications of our results to understand the origins of *T*. *cruzi* and Chagas disease.

## Methods

### Ethics statement

Permits for field research, allowing handling and euthanizing bats, were granted by the Ministerio del Ambiente de Ecuador (002–07 IC-FAU-DNBAPVS/MA, and 008-IC-INSEC-DPL-MA). Bats were euthanized in the field by thoracic compression in 2007 and by inhalation of carbon dioxide in 2012. The methods we used for manipulation and euthanizing of bats are in agreement with the guidelines of the American Society of Mammalogists for the use of wild mammals in research [20]. No protocol of an institutional animal care and use committee (IACUC) was required for this research because Pontificia Universidad Católica del Ecuador does not have an IACUC.

### Field surveys of bats and triatomines in Ecuador

A total of 74 bats were caught during January 2007 and July 2012 using a butterfly net at roosts inside human constructions or by mist netting forest patches in the following rural communities: Bellamaría Chica, Bellamaría, Chaquizhca and Chinguilamaca in Loja province, and Rancho Alegre in Zamora Chinchipe province. Additionally, at the Chinguilamaca and Rancho Alegre locations, opportunistic triatomine insect searches within the bat roosts were performed manually. Taxonomic identification of bats and triatomines was conducted by morphological examination following [21] and [22], respectively.

Captured bats were euthanized; samples of blood, liver, heart, and muscle tissues were collected, and from the animals captured in 2012, blood smears and trypanosome cultures were also prepared. A blood aliquot of 150μl was inoculated in biphasic Novy-Nicolle-MacNeal (NNN) culture and the growth of parasites was tracked weekly by microscopy during the first month, and then again at three and six months after initial inoculation. Positive samples containing *Trypanosoma*-like parasites were grown until counts reached more than 100 parasites per field and were then transferred to Liver Infusion Tryptose (LIT) medium for further growth.

### Detection of trypanosomes

Direct microscopic detection of trypanosomes was performed on the animals captured in 2012. DNA was extracted from samples of blood, liver, muscle, or positive cultures with the DNeasy kit (Qiagen, Valencia, CA) following manufacturer´s protocol. Samples were PCR amplified with the primer sets S35/S36 and 121/122 to detect infections with *T*. *cruzi* and *T*. *rangeli*, respectively [23–26]. Products were visualized on 2% agarose gels. No triatomines were analyzed because of problems with extracting DNA from improperly preserved specimens.

### Phylogenetic identification of trypanosome lineages

We sequenced fragments of the 18S rRNA and cytb genes of *Trypanosoma* from a subset of eight positive animals from three localities that yielded positive results (S1 Table). Not all the positive samples were sequenced because animals were collected in groups and most likely share the same parasite genotypes. To amplify the 18S rRNA, we followed a nested PCR protocol [27] with the following modifications: the initial PCR amplification was conducted with the newly designed primers SSU4_F (GTGCCAGCACCCGCGGTAAT) and 18Sq1R (CCACCGACCAAAAGCGGCCA); both nested PCR amplifications were run with a touchdown PCR profile [28]. The cytb gene was amplified following a published protocol [16]. After cleaning the PCR products with ExoSAP-IT (Affymetrix, Santa Clara, CA), we did sequencing reactions in both directions with the ABI BigDye chemistry (Applied Biosystems, Inc., Foster City, CA), and sequenced the fragments on an ABI 3730xl DNA Analyzer automatic sequencer (Applied Biosystems, Inc., Foster City, CA).

We built a matrix for each gene with sequences of previous studies of lineage diversity of *T*. *cruzi* e.g., [3,5,29,30] and also used sequences of *Trypanosoma dionisii* and *Trypanosoma erneyi* as outgroups [31] (S1 Dataset). We assembled each gene fragment with the Geneious Alignment tool in Geneious v. 6.1.8 [32], and the alignments were checked and corrected manually. The 18S rRNA and cytb alignments were cropped at 863 bp (99 sequences) (S2 Dataset) and 490 bp (362 sequences) (S3 Dataset) respectively. We built a network genealogy for the 18S rRNA gene with the program SplitsTree v. 4.11.3 using the NeighborNet method [33]. Internode supports were estimated by performing 100 bootstrap replicates using the same parameters optimized for network inferences. For the alignment of the cytb gene we started a maximum likelihood run in RAxML v. 8 [34] and interrupted it after obtaining the reduced matrix that contains only one sequence per unique haplotype (S4 Dataset). Following this, we ran to completion the analysis with the GTR-CAT approximation on the reduced matrix. The GTR-CAT approximation is a rapid algorithm for ML analyses that resembles the GTR-G model, but it is optimized for faster performance [35].

Also, to test the combinability of the 18S rRNA and cytb genes for concatenated phylogenetic analysis we used the software MLSTest [36] to run the analysis ILD-BIONJ [37] that is an efficient variant of the incongruence length difference test [38]. We ran ILD-BIONJ with reduced alignments that only contained strains represented for both genes. This analysis determined that our loci had significantly different branching patterns (p = 0.0099), so no further concatenated analyses were performed.

### Comparisons of nucleotide diversity among *T*. *cruzi* lineages

We calculated the nucleotide diversity (π) of each *T*. *cruzi* subdivision on the cytb tree (i.e., TcI, TcII, TcIII-TcVI, Tcbat, and the subspecies *T*. *c*. *marinkellei*). In Mega v. 6 [39] we used the option “compute mean diversity in entire population”, which calculates a nucleotide diversity index that is independent of sample size (equation 12.73 in [40]). Standard errors were estimated by 1,000 bootstrap replicates. The 18S rRNA fragment was not used for these calculations because of indels within the alignment, which are removed from the calculations producing severe underestimations of nucleotide diversity.

## Results

### Detection of trypanosomes

Of the 74 bats examined, 27 (36.5%) were positive for *T*. *cruzi*, and only one was positive for *T*. *rangeli*. The species with the highest infection rate was *Artibeus fraterculus* (90.1%), while *Myotis* sp. and *Glossophaga soricina* were positive in one locality each (Table 1). All positive bats were negative by direct microscopy, and trypanosomes were detected only by PCR.

**Table 1:** Results of PCR screenings for *Trypanosoma cruzi* and *Trypanosoma rangeli*.

Bat species	Locality	Bats examined	*T*. *cruzi*	*T*. *rangeli*
*Artibeus fraterculus*	Bella Maria Chica, Loja	22	20 (90.1%)	0
*Desmodus rotundus*	Bella Maria, Loja	7	3 (42.9%)	1 (14.3%)
*Glossophaga soricina*	Bella Maria, Loja	5	0	0
*Glossophaga soricina*	Chaquizhca, Loja	12	3 (25%)	0
*Molossus molossus*	Chinguilamaca, Loja	3	0	0
*Myotis sp*.	Chinguilamaca, Loja	18	1 (5.6%)	0
*Myotis sp*.	Rancho Alegre, Zamora	7	0	0
5 species	5 localities	74 bats	27 (36.5%)	1 (1.6%)

### Phylogenetic identification of trypanosome lineages

**Fig 1:**
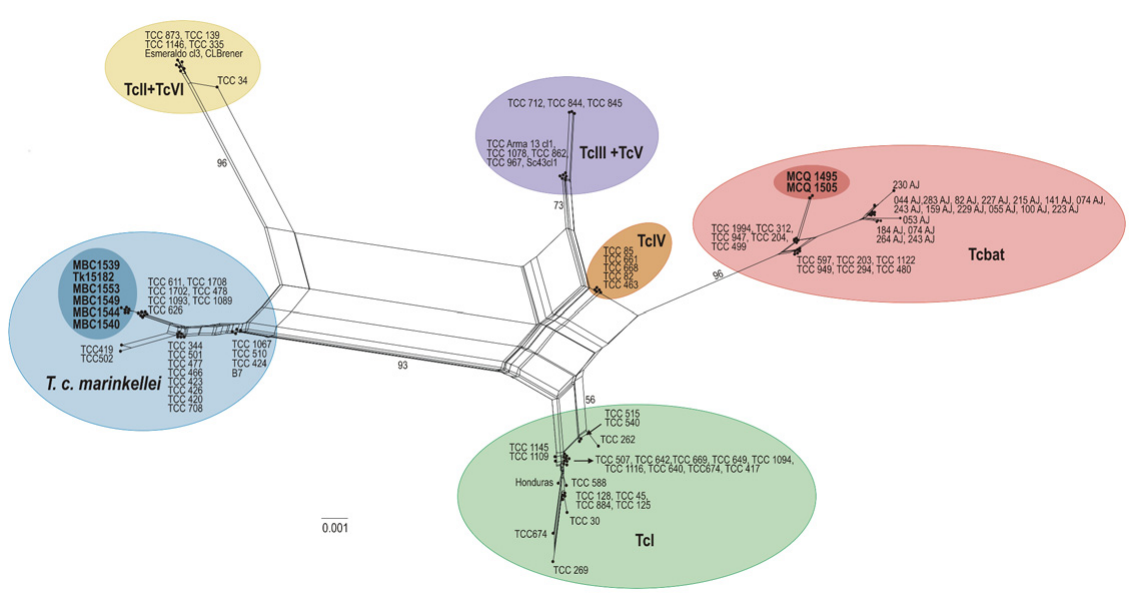
Network genealogy using partial 18S rRNA gene sequences from eight new trypanosomes characterised in this study (in bold) plus 70 other sequences from all DTUs (TcI-TcVI and Tcbat) of *T*. *cruzi* and 22 sequences from *T*. *c*. *marinkellei*. Network constructed with the NeighborNet algorithm excluding all conserved sites and with uncorrected p-distance. Numbers in nodes correspond to bootstrap support values using the same parameter optimized for network inferences.

**Fig 2:**
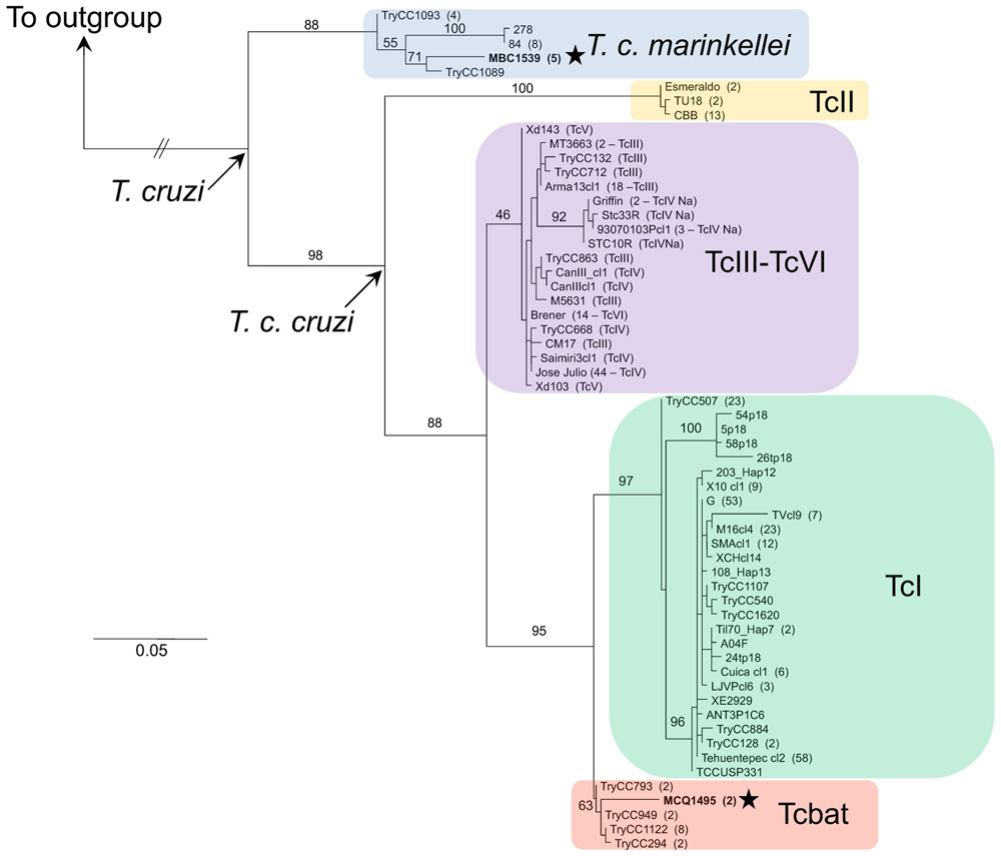
Mitochondrial phylogeny of *Trypanosoma cruzi*. Maximum likelihood tree of a fragment of the cytb gene of *Trypanosoma cruzi* and *T*. *dionisii* as outgroup, representing 60 haplotypes from 362 sequences. The parentheses at the tip labels contain the number of identical sequences per each haplotype, and in the TcIII-TcVI group the DTU identity for each haplotype is indicated. Numbers on branches correspond to bootstrap support values. Stars indicate the haplotypes found in Ecuador.

Sequences of the 18S rRNA and cytb genes were obtained from seven out of the eight positive samples; for one sample (TK 151852), only the 18S rRNA fragment was successfully acquired. Six out the eight samples were found infected with *T*. *cruzi marinkellei*, and two were positive for Tcbat (S1 Table). The network genealogy of the 18S rRNA gene resulted in distinctive clusters for each genetic lineage (Fig 1). The phylogenetic analysis of the cytb gene revealed five mitochondrial lineages within *T*. *cruzi* sensu lato, differing from the traditional DTU classification by clustering in a single clade the DTUs TcIII, TcIV, TcV, and TcVI (Fig 2).

### Comparisons of nucleotide diversity among *T*. *cruzi* lineages

**Fig 3:**
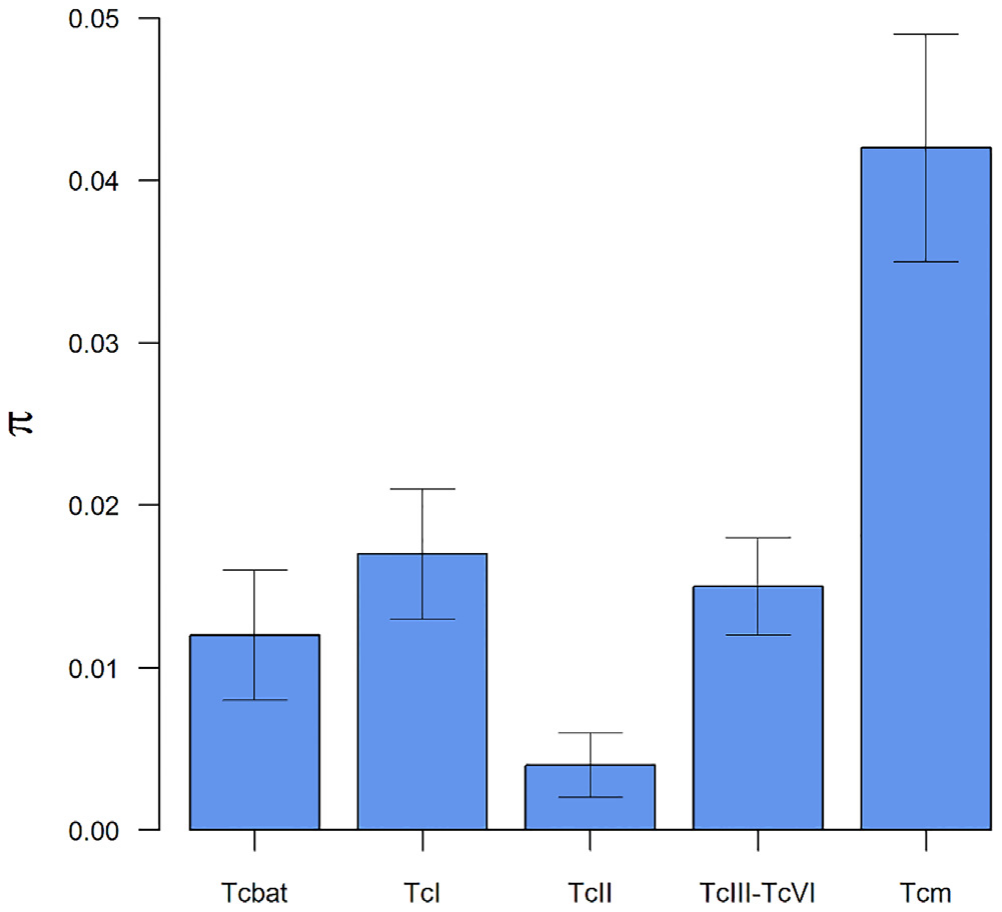
Mitochondrial diversity of *Trypanosoma cruzi*. Nucleotide diversity (π) of mitochondrial lineages of *Trypanosoma cruzi* calculated for the haplotypes of the cytb gene. The subspecies *T*. *c*. *marinkellei* shows larger nucleotide diversity than the other examined lineages. Whiskers in each bar indicate the standard error.

Among the mitochondrial lineages, *T*. *c*. *marinkellei* shows the largest nucleotide diversity in the cytb gene, followed in descending order by TcI, TcIII-TcVI, Tcbat, and TcII. The nucleotide diversity within the *T*. *c*. *marinkellei* is 2.6 times larger than in TcI (0.042 vs. 0.017), and Tcbat nucleotide diversity (0.012) is comparable with the diversity of the pooled DTUs TcIII-TcVI (0.015) (Fig 3).

### Triatomine vectors associated with bats

**Fig 4:**
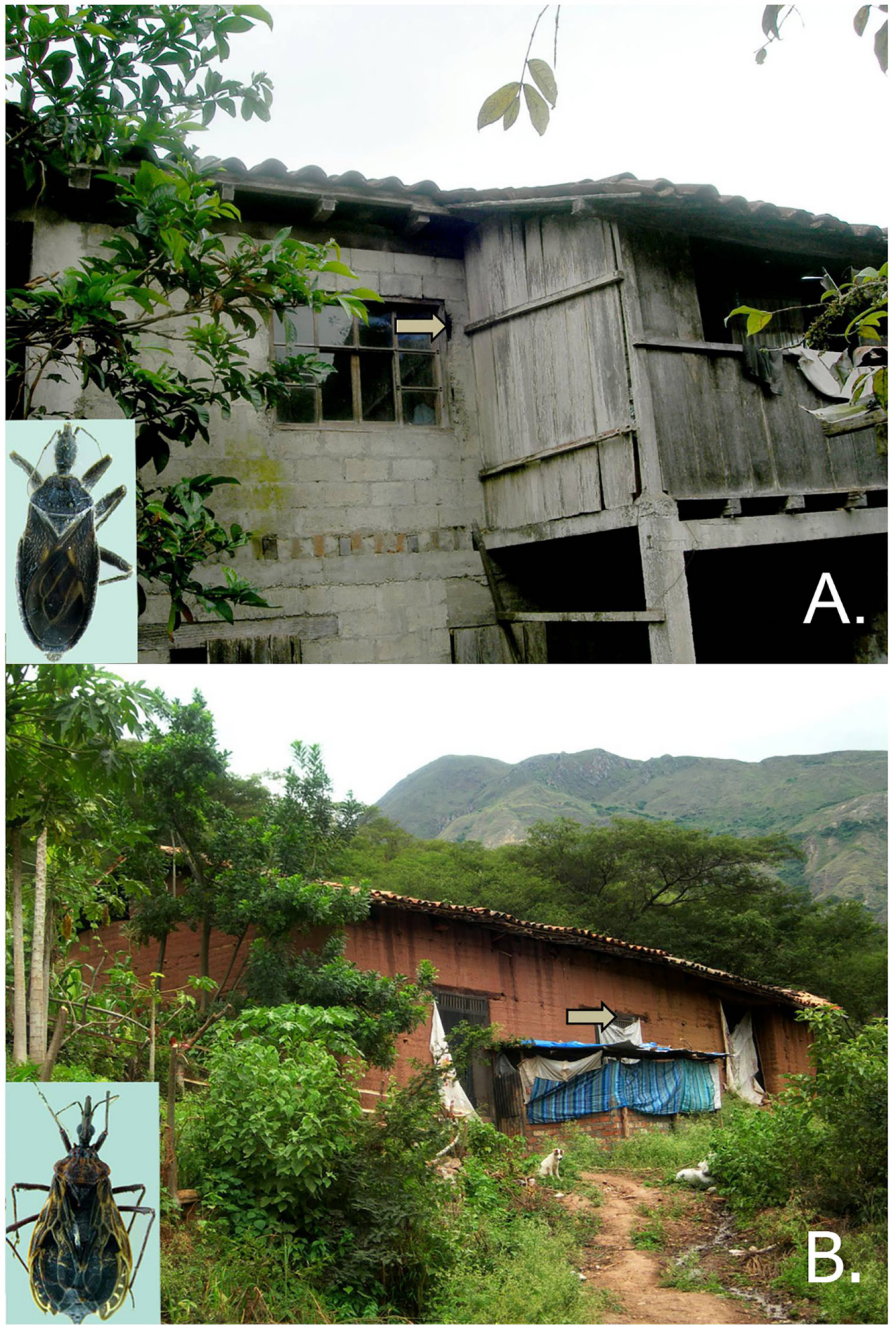
Constructions in Ecuador where *Cavernicola pilosa* and *Triatoma dispar* were found in association with bats. **A.** House with cinderblock walls at Rancho Alegre, Zamora Chinchipe where *C*. *pilosa* (inset) was inhabiting a roost of *Myotis* sp. **B.** Adobe barn in Chinguilamaca, Loja where *T*. *dispar* (inset) was found associated with *Molossus molossus* and *Myotis* sp. Arrows indicate the entrances to the bat roosts where the insects were collected.

Triatomine bugs were found in two bat roosting sites. One live adult *Cavernicola pilosa* associated with *Myotis* sp. was found inside the walls of a two-story cinderblock house in Barrio Rancho Alegre, close to the town of Zamora, Zamora Chinchipe Province (WGS84, 78.86497°W, 03.98589°S; 885 m) (Fig 4). One live adult of *Triatoma dispar* was found associated with *Molossus molossus* and *Myotis* sp. in the window crevice of an adobe barn that is used to raise chickens. One of the *Myotis* sp. was positive for *T*. *c*. *marinkellei*. This barn is close to a two-story house located in Chinguilamaca, Loja Province (WGS84, 79.32769°W, 04.18938°S; 1358 m) (Fig 4).

## Discussion

The large genetic diversity for the bat lineages of *T*. *cruzi* sensu lato (i.e., Tcbat and *T*. *c*. *marinkellei*), together with the phylogenetic pattern of bat-exclusive species basal to *T*. *cruzi* (e.g., *T*. *dionisii*, *T*. *erneyii*), strongly suggest a long evolutionary history between these parasites and bats; a history that is longer and more complex than with any other group of *T*. *cruzi* reservoir hosts (e.g., didelphid marsupials). This may indicate that bats are the original reservoir hosts of *T*. *cruzi*, as it has been suggested in the bat-seeding hypothesis [17,18]. Similar rationale has been implemented in deciphering the origins of the malaria parasite *Plasmodium falciparum* in non-human African apes; *P*. *falciparum* has higher nucleotide diversity in chimpanzees than in humans [41], and human parasites nest within gorilla lineages [42].

*Trypanosoma cruzi* seems to have switched from bats to non-volant mammals more than three million years ago [43], with humans among the most recent hosts of this parasite, since the peopling of the Americas occurred less than 15 thousand years ago [44]. Thus, the origins of Chagas disease can be explained in two series of host switching events. First, a single host switch likely occurred from bats to non-volant mammals; this should have been facilitated by generalist triatomines able to interact with bats and non-volant mammals, such as some *Panstrongylus* and *Triatoma* species [22]. Second, several host switches of *T*. *cruzi* from bats and wild, non-volant mammals to humans are required to explain the diversity of lineages circulating in human populations, since all DTUs have diverged earlier than the arrival of humans to the Americas [43]. These host switches to humans could have occurred directly from wild triatomine populations during the human colonization of the Americas, since the domestication process of triatomines occurred several times and likely over extensive periods of time [45].

The findings of Tcbat and *T*. *c*. *marinkellei* in southern Ecuador greatly extend the known distribution of these lineages. Tcbat has been previously known from few localities in Brazil, Colombia and Panamá [3,5,6,15]; *T*. *c*. *marinkellei* has been reported in Bolivia, Brazil, Colombia, Panamá, and Venezuela e.g., [2,13,19,29,30,46]. Most of these records are from South America, east of the Andes, thus these Ecuadorian records from west of the Andes, could prove useful to track the biogeographic history and dispersal patterns of these trypanosomes. Nonetheless, it is surprising that in surveys of bat trypanosomes in Venezuela and Bolivia, Tcbat has not been detected [30,47], suggesting that this lineage may have a wide but patchy distribution, with locally high prevalences.

Previously, Tcbat has been reported in association with nine bat species that are either frugivorous or insectivorous and belong to the families Emballonuridae, Noctilionidae, Phyllostomidae, and Vespertilionidae [3,5,6,48]. Our findings of Tcbat in the phyllostomid bat *Glossophaga soricina*, a mostly nectar feeding species, increase the breadth of dietary guilds of hosts and reaffirms the host-generalist condition among bats of this *T*. *cruzi* lineage. On the other hand, *T*. *c*. *marinkellei* has been detected in at least 11 species of only phyllostomid bats [2,13,19,29,30,46]. The positive animals reported here add new host species—*A*. *fraterculus* and *Myotis* sp.—to *T*. *c*. *marinkellei*.

In addition, *Triatoma dispar* could be a second potential vector of *T*. *c*. *marinkellei*, since the trapped individual was found in the roosting site were *Myotis* sp. was captured. Previously, only *C*. *pilosa* was thought to be a vector of *T*. *c*. *marinkellei* following the observations of Marinkelle [12]. The paucity in triatomine searches in bat roosts may be responsible for the lack of information on vectors of the bat lineages of *T*. *cruzi*. There are several sylvatic species of triatomines, some already reported in association with bats (e.g., *Eratyrus cuspidatus*, *E*. *mucronatus*, *Triatoma rubida*, *Panstrongylus geniculatus*) [22], but new surveys are required to try to isolate trypanosomes from these triatomines and their bat hosts.

Not surprisingly, PCR detection was more sensitive than direct microscopy [49]. PCR detection is highly encouraged to detect trypanosomes from preserved tissues and in degraded or difficult samples e.g., [50–52]. Although the assays with primers S35/S36 and 121/122 work generally well, we recommend amplification and sequencing of a fragment of the 18S rRNA gene (see materials and methods), because the amplification bands are bright and easy to visualize, and other trypanosomes species could be detected (e.g., [19,27]).

The *T*. *cruzi* prevalence of 36.5% in bats with 90.1% prevalence in *Artibeus fraterculus* found in this study is unusually high. Previous *T*. *cruzi* surveys in similar environments in Ecuador detected lower prevalences in non-volant mammals [53,54], indicating that transmission cycles involving bats could be more active than those in rodents and marsupials in this region. Some life-history traits make bats ideal long-term reservoir hosts of pathogens: bats are long-lived animals, are able to use different shelters, and can live in large aggregations that might attract vectors and facilitate transmission [55]. In particular, *A*. *fraterculus* in Loja province are resilient to human disruption of the environment, live in large aggregations, and use man-made structures as roosts [56,57]. Further studies are required to determine the ecological variables (i.e., triatomine vectors) associated with the high prevalence of *T*. *cruzi* in bats.

The triatomines associated with bats reported herein increase the number of the species previously considered to be restricted to sylvatic environments but which now are also known to occur in human environments. Previously, *Cavernicola pilosa* and *Triatoma dispar* had been found only in sylvatic conditions and associated with wild mammals [22,58], with exception of one report of a *C*. *pilosa* on a house roof [59], and a recent report of individuals of *T*. *dispar* in domestic areas [60]. *C*. *pilosa* has been reported in roost sites of at least nine bat species within five families [22,58]. *Triatoma dispar* has been reported associated with the sloth *Choloepus hoffmanni* [22,61]. Our findings are the first records of *C*. *pilosa* and *T*. *dispar* associated with vespertilionid bats (*Myotis* sp.), and *T*. *dispar* associated to a molossid bat (*Molossus molossus*).

The findings of *C*. *pilosa* and *T*. *dispar* within human constructions may be attributed as accidental colonization, because of our failure to find established colonies with eggs and nymphs. Nonetheless, bats roost in deep cracks in the walls and roofs, making the collecting process difficult, and therefore bat roosts may be systematically overlooked in triatomine surveys inside houses. Despite recent intensive triatomine surveys in Ecuador, this is the first report of *T*. *dispar* in Loja province [62–64]. The association of *C*. *pilosa* and *T*. *dispar* with bats roosting in human constructions is remarkable as it may have human health implications. Tcbat has been reported in bats, and recently in association with humans [14,15]. Potentially, triatomines associated with bats might opportunistically feed on and be able to transmit trypanosomes to humans. The triatomines reported in this study were collected in constructions with characteristics associated with risk factors for *T*. *cruzi* infections (Fig 4); living in houses with adobe walls in Loja province or houses with open and mixed walls in the Ecuadorian Amazon have been identified as risk factors for anti-*T*. *cruzi* seropositivity in human populations [65,66].

The high nucleotide diversity of *T*. *c*. *marinkellei* supports a long evolutionary relationship between *T*. *cruzi* and bats, implying that bats are the original hosts of this important parasite. Tcbat and *T*. *c*. *marinkellei* are for the first time recorded in Ecuador, expanding their distribution in South America to the western side of the Andes, demonstrating that both lineages are widely distributed in South America. Also, we suggest potential vectors for these two genetic lineages of trypanosomes associated with bats in rural areas of southern Ecuador.

## Supporting Information

S1 Table Ecuadorian samples sequenced for this study. Trypanosome-positive samples selected for DNA sequencing with respective GenBank accession numbers, locality, bat species, and whether trypanosome sequences were obtained from LIT cultures or directly from liver tissue.
(DOCX)

S1 Dataset GenBank accession numbers. 18S rRNA and cytb sequences used in this study with corresponding GenBank accession numbers within brackets. Codes in bold indicate the sequences newly generated for this research.
(DOCX)

S2 Dataset Alignment of a fragment of the 18S rRNA gene. Alignment of an 806 bp fragment of the 18S rRNA gene from 100 sequences of *Trypanosoma cruzi* sensu lato. This file was used for the network genealogy (Fig 2).
(TXT)

S3 Dataset Alignment of 362 sequences of the cytb gene. Alignment of a 490 bp fragment of the cytb gene from 361 sequences of *Trypanosoma cruzi* and one *T*. *dionisii*.
(TXT)

S4 Dataset Alignment of 60 haplotypes of the cytb gene. A subset of the alignment of Additional file 3 containing one sequence per each unique haplotype. This file was used for the cytb maximum likelihood tree and estimations of nucleotide diversity (Fig 3).
(TXT)
