# Supplementary material for: Bats, Trypanosomes, and Triatomines in Ecuador: New Insights into the Diversity, Transmission, and Origins of Trypanosoma cruzi and Chagas Disease
Source: PLoS One. 2015 Oct 14;10(10):e0139999. doi: 10.1371/journal.pone.0139999 (PMC4605636; doi:10.1371/journal.pone.0139999)
Supplement: S1 Table — Trypanosome-positive samples selected for DNA sequencing with respective GenBank accession numbers, locality, bat species, and whether trypanosome sequences were obtained from LIT cultures or directly from liver tissue. (DOCX) [file pone.0139999.s001.docx]

| **Bat code** | **Locality** | **Bat species** | **DNA Source** | **Trypanosome lineage** | **18S rRNA** | **cytb** |
| --- | --- | --- | --- | --- | --- | --- |
| MBC_1539 | Bella Maria Chica, Loja | *Artibeus fraterculus* | LIT culture | *T. c. marinkellei* | KT829452 | KT829468 |
| MBC_1540 | Bella Maria Chica, Loja | *Artibeus fraterculus* | LIT culture | *T. c. marinkellei* | KT829453 | KT829469 |
| MBC_1544 | Bella Maria Chica, Loja | *Artibeus fraterculus* | LIT culture | *T. c. marinkellei* | KT829454 | KT829470 |
| MBC_1549 | Bella Maria Chica, Loja | *Artibeus fraterculus* | LIT culture | *T. c. marinkellei* | KT829455 | KT829471 |
| MBC_1553 | Bella Maria Chica, Loja | *Artibeus fraterculus* | LIT culture | *T. c. marinkellei* | KT829456 | KT829472 |
| TK151852 | Chinguilamaca, Loja | *Myotis* sp. | Liver tissue | *T. c. marinkellei* | KT829457 | -- |
| MCQ_1495 | Chaquizhca, Loja | *Glossophaga soricina* | LIT culture | Tcbat | KT829450 | KT829466 |
| MCQ_1505 | Chaquizhca, Loja | *Glossophaga soricina* | LIT culture | Tcbat | KT829451 | KT829467 |
