# Supplementary material for: Bats, Trypanosomes, and Triatomines in Ecuador: New Insights into the Diversity, Transmission, and Origins of Trypanosoma cruzi and Chagas Disease
Source: PLoS One. 2015 Oct 14;10(10):e0139999. doi: 10.1371/journal.pone.0139999 (PMC4605636; doi:10.1371/journal.pone.0139999)
Supplement: S1 Dataset — 18S rRNA and cytb sequences used in this study with corresponding GenBank accession numbers within brackets. Codes in bold indicate the sequences newly generated for this research. (DOCX) [file pone.0139999.s002.docx]

**18S rRNA**

***Trypanosoma cruzi marinkellei:* MBC_1539 [GenBank: KT829452]; MBC_1540 [GenBank: KT829453]; MBC_1544 [GenBank: KT829454]; MBC_1549 [GenBank: KT829455]; MBC_1553 [GenBank: KT829456]; TK151852 [GenBank: KT829457];** B3 [GenBank: FJ649484]; B7 [GenBank: AJ009150]; CBT10 [GenBank: JX845157]; CBT11 [GenBank: JX845158]; CBT19 [GenBank: JX845159]; CBT3 [GenBank: JX845151]; CBT32 [GenBank: JX845160]; CBT33 [GenBank: JX845161]; CBT35 [GenBank: JX845162]; CBT36 [GenBank: JX845163]; CBT4 [GenBank: JX845152]; CBT5 [GenBank: JX845153]; CBT7 [GenBank: JX845154]; CBT8 [GenBank: JX845155]; CBT9 [GenBank: JX845156]; Mor61 [GenBank: JX845164]; Mor86 [GenBank: JX845165]; Pomor24 [GenBank: JX845166]; TCCUSP1067 [GenBank: FJ001649]; TCCUSP332 [GenBank: FJ001635]; TCCUSP344 [GenBank: FJ001664]; TCCUSP420 [GenBank: FJ001636]; TCCUSP421 [GenBank: FJ001637]; TCCUSP423 [GenBank: FJ001638]; TCCUSP424 [GenBank: FJ001639]; TCCUSP426 [GenBank: FJ001640]; TCCUSP466 [GenBank: FJ001641]; TCCUSP477 [GenBank: FJ001642]; TCCUSP478 [GenBank: FJ001643]; TCCUSP485 [GenBank: FJ001644]; TCCUSP494 [GenBank: FJ001645]; TCCUSP501 [GenBank: FJ001665]; TCCUSP510 [GenBank: FJ001646]; TCCUSP626 [GenBank: FJ001647]; TCCUSP708 [GenBank: FJ001648]; **TryCC1067 [GenBank: KT829463]**; TryCC1089 [GenBank: EU867808]; TryCC1093 [GenBank: EU867809]; TryCC1093 [GenBank: EU867809]; TryCC1702 [GenBank: FJ900244]; TryCC1708 [GenBank: FJ900245]; **TryCC419 [GenBank: KT829465]; TryCC502 [GenBank: KT829464]**; TryCC611 [GenBank: FJ900243].

**Tcbat:**  **MCQ_1495 [GenBank: KT829450]; MCQ_1505 [GenBank: KT829451]**; 044_AJ_Guanabano_1 [GenBank: JQ965534]; 053_AJ_Guanabano [GenBank: JQ965548]; 055_AJ_Guanabano [GenBank: JQ965537]; 074_AJ_Guanabano [GenBank: JQ965542]; 082_AJ_Bohio_1 [GenBank: JQ965532]; 100_AJ_Guava [GenBank: JQ965541]; 141_AJ_Gigante [GenBank: JQ965540]; 159_AJ_Gigante [GenBank: JQ965546]; 184_AJ_Gigante_1 [GenBank: JQ965539]; 215_AJ_Guava [GenBank: JQ965538]; 223_AJ_Guava [GenBank: JQ965545]; 227_AJ_Guava [GenBank: JQ965536]; 229_AJ_Guava [GenBank: JQ965535]; 230_AJ_Guava [GenBank: JQ965547]; 243_AJ_Guava [GenBank: JQ965544]; 264_AJ_Leon [GenBank: JQ965543]; 283_AJ_Leon [GenBank: JQ965533]; TryCC1122 [GenBank: FJ001628]. ; TryCC1994 [GenBank: FJ001634]; TryCC203 [GenBank: FJ001617]; TryCC204 [GenBank: FJ001618]; TryCC294 [GenBank: FJ001619]; TryCC312 [GenBank: FJ001620]; TryCC480 [GenBank: EU867804]; TryCC499 [GenBank: FJ001622]; TryCC597 [GenBank: FJ001623]; TryCC793 [GenBank: FJ001634]; TryCC947 [GenBank: FJ001626]; TryCC949 [GenBank: FJ001627].

**TcI:** TryCC417 [GenBank: FJ001631]**;** AEAAB [GenBank: AY491763]; AM-ANV [GenBank: EU755222]; AR5P [GenBank: FJ183394]; AV-AAF [GenBank: EU755221]; DRS [GenBank: FJ549378]; EP24X [GenBank: FJ549371]; EP31P [GenBank: FJ549372].; G [GenBank: AF239981]; Honduras [GenBank: AY785581]; PAN ma1 [GenBank: FJ549381]; Roma 06 [GenBank: FJ549375]; TryCC1108 [GenBank: FJ549382]; TryCC1109 [GenBank: EU867806]; TryCC1116 [GenBank: EU867807]; **TryCC1145 [GenBank: KT829460]**; TryCC507 [GenBank: FJ001632]; **TryCC515 [GenBank: KT829458]**; TryCC540 [GenBank: FJ555616]; **TryCC588 [GenBank: KT829459]**; TryCC640 [GenBank: FJ001633]; TryCC642 [GenBank: FJ001624]; TryCC649 [GenBank: EU755225]; TryCC669 [GenBank: EU755227]; **TryCC674 [GenBank: KT829461];** TryCC884 [GenBank: FJ549377]; XE6863/3 [GenBank: FJ549379].

**TcII:** Y [GenBank: AF301912]**;** 573LU [GenBank: FJ001625]; Basileu [GenBank: FJ001629]; Esmeraldo cl3 [GenBank: AY785564]; IB42X [GenBank: FJ001616]; Peru [GenBank: X53917]; Sinesio [GenBank: FJ001621]; **TryCC873 [GenBank: KT829462]**.

**TcIII:** MT3869 [GenBank: AF303660]**;** Arma 13 cl1 [GenBank: FJ549385]; MS2682 [GenBank: EU755230].; MT3663 [GenBank: AF288660]; QJIII [GenBank: FJ549380]; TryCC844 [GenBank: AF303660]; TryCC862 [GenBank: FJ183397]; TryCC863 [GenBank: FJ549376].

**TcIV:** Jose Julio [GenBank: AY491761]**;** MS2440 [GenBank: EU755224]; TryCC661 [GenBank: EU755226]; TryCC668 [GenBank: FJ183396]; TryCC698 [GenBank: EU755228]; TryCC82 [GenBank: EU755217]; TryCC82 [GenBank: EU755218].

**TcV:** TryCC967 [GenBank: AF228685]; Sc43cl1 [GenBank: AF228685].

**TcVI:** CL Brener [GenBank: AF245383].

***Trypanosoma dionisii:*** TryCC1110 [GenBank: FJ001662]***;*** TryCC495 [GenBank: FJ001667]; TryCC1147 [GenBank: FJ001663].

***Trypanosoma erneyii:*** TryCC1946 [GenBank: JN040989].

**Cytb**

***Trypanosoma cruzi marinkellei:* MBC_1539 [GenBank: KT829468]; MBC_1540 [GenBank: KT829469]; MBC_1544 [GenBank: KT829470]; MBC_1549 [GenBank: KT829471]; MBC_1553 [GenBank: KT829472];** 225 [GenBank: JN651285]; 232 [GenBank: JN651286]; 24 [GenBank: JN651278]; 26 [GenBank: JN651279]; 27 [GenBank: JN651280]; 278 [GenBank: JN651284]; 79 [GenBank: JN651281]; 80 [GenBank: JN651282]; 82 [GenBank: JN651283]; 84 [GenBank: JN651287]; M1117 [GenBank: AJ130927]; TCCUSP1067 [GenBank: FJ900247]; TCCUSP344 [GenBank: JN543701]; TCCUSP494 [GenBank: FJ900246]; TCCUSP501 [GenBank: JN543702]; TryCC 1093 [GenBank: FJ002262]; TryCC1089 [GenBank: FJ900248]; TryCC1093 [GenBank: FJ002262].

**Tcbat: MCQ_1495 [GenBank: KT829466]; MCQ_1505 [GenBank: KT829467];** 13 [GenBank: KC951627]; 2 [GenBank: KC951626]; FBN45 [GenBank: KC951624]; NY13 [GenBank: KC951625]; SL2643 [GenBank: KC951623]; SL77 [GenBank: KC951621]; SLBAT1 [GenBank: KC951622]; TryCC1122 [GenBank: FJ002261]; TryCC203 [GenBank: FJ002253]; TryCC294 [GenBank: FJ002254]; TryCC597 [GenBank: FJ002257]; TryCC793 [GenBank: FJ002258]; TryCC947 [GenBank: FJ002259]; TryCC949 [GenBank: FJ002260].

**TcI:** 100 [GenBank: JX431268]; 10462P2C3 [GenBank: JX431281]; 10462P2C7 [GenBank: JX431282]; 108_Hap13 [GenBank: JF267940]; 10968P1C1 [GenBank: JX431283]; 113 [GenBank: JX431269]; 11541 [GenBank: JQ581335]; 116 [GenBank: JX431270]; 11713 [GenBank: JQ581336]; 11804 [GenBank: JQ581337]; 154 [GenBank: JX431271]; 203_Hap12 [GenBank: JF267939]; 24tp18 [GenBank: EU559324]; 26tp18 [GenBank: EU559326]; 38 [GenBank: JX431260]; 46 [GenBank: JX431261]; 54p18 [GenBank: EU559328]; 58p18 [GenBank: EU559327]; 5p18 [GenBank: EU559329]; 66 [GenBank: JX431262]; 67 [GenBank: JX431263]; 70 [GenBank: JX431264]; 71 [GenBank: JX431265]; 83 [GenBank: JX431266]; 92090802Pcl1 [GenBank: JQ581332]; 95 [GenBank: JX431267]; A04F [GenBank: EU559323]; AAA7cl15 [GenBank: HQ713738]; AAB3cl2 [GenBank: HQ713707]; AAC1cl3 [GenBank: HQ713697]; AACf1cl2 [GenBank: HQ713746]; AAD6cl7 [GenBank: HQ713698]; AADm1cl1 [GenBank: HQ713688]; AM-ANV [GenBank: EU856370]; ANITAII [GenBank: JX431272]; ANT3P1C6 [GenBank: JX431284]; AR5P [GenBank: FJ183398]; AV-AAF [GenBank: EU856369]; B2085 [GenBank: JQ581339]; CAM6 [GenBank: JX431273]; Cepa2cl6 [GenBank: HQ713709]; COTMA38 [GenBank: JQ581340]; Coy11cl15 [GenBank: HQ713737]; CRISTY [GenBank: JX431274]; Cuica cl1 [GenBank: AJ439719]; D16cl8 [GenBank: HQ713680]; D18cl10 [GenBank: HQ713679]; D1cl14 [GenBank: HQ713696]; D5cl7 [GenBank: HQ713694]; DAVIS990cl1 [GenBank: JQ581341]; Dm11cl7 [GenBank: HQ713692]; Dm38cl16 [GenBank: HQ713693]; Dm7cl6 [GenBank: HQ713695]; DRS [GenBank: FJ599394]; EHcl1 [GenBank: KF220728]; EHcl2 [GenBank: KF220727]; EHcl3 [GenBank: KF220725]; EHcl4 [GenBank: KF220729]; EHcl5 [GenBank: KF220726]; EP24X [GenBank: FJ549386]; EP31P [GenBank: FJ549387]; FCHcl1 [GenBank: KF220710];FCHcl15 [GenBank: KF220714]; FCHcl2 [GenBank: KF220711]; FCHcl3 [GenBank: KF220712]; FCHcl4 [GenBank: KF220713]; G [GenBank: FJ156759]; Gal61cl16 [GenBank: HQ713689]; GCcl1 [GenBank: KF220750]; GCcl2 [GenBank: KF220751]; GCcl3 [GenBank: KF220752]; GCcl4 [GenBank: KF220753]; GCcl5 [GenBank: KF220754]; H10cl10 [GenBank: HQ713747]; Hap1 [GenBank: JF267928]; Hap10 [GenBank: JF267937]; Hap11 [GenBank: JF267938]; Hap3 [GenBank: JF267930]; IM4810 [GenBank: JQ581342]; LERcl1 [GenBank: KF220720]; LERcl12 [GenBank: KF220723]; LERcl14 [GenBank: KF220724]; LERcl15 [GenBank: KF220721]; LERcl16 [GenBank: KF220722]; LJVPcl10 [GenBank: KF220744]; LJVPcl6 [GenBank: KF220741]; LJVPcl7 [GenBank: KF220740]; LJVPcl8 [GenBank: KF220742]; LJVPcl9 [GenBank: KF220743]; M13 [GenBank: JQ581344]; M16cl4 [GenBank: JQ581346]; M18 [GenBank: JQ581345]; M7 [GenBank: JQ581343]; MICH1 [GenBank: JX431275]; N10 [GenBank: KC951575]; N11 [GenBank: KC951576]; N13 [GenBank: KC951577]; N17 [GenBank: KC951578]; N2 [GenBank: KC951579]; N22 [GenBank: KC951583]; N3134 [GenBank: KC951585]; N32423 [GenBank: KC951584]; N5P14cl17 [GenBank: HQ713715]; N7 [GenBank: KC951580]; N8 [GenBank: KC951581]; N9 [GenBank: KC951582]; NA3cl4 [GenBank: HQ713713]; NB2cl15 [GenBank: HQ713712]; NC2cl8 [GenBank: HQ713714]; NCHcl1 [GenBank: KF220735]; NCHcl2 [GenBank: KF220736]; NCHcl3 [GenBank: KF220737]; NCHcl4 [GenBank: KF220738]; NCHcl5 [GenBank: KF220739]; NDm1cl7 [GenBank: HQ713686]; Necoclicl6 [GenBank: HQ713711]; NINOA [GenBank: JX431276]; NR1cl10 [GenBank: HQ713685]; P234 [GenBank: JQ581347]; P238 [GenBank: JQ581348]; P268 [GenBank: JQ581349]; PALDA20 [GenBank: JQ581353]; PALDA21 [GenBank: JQ581354]; PALDA4 [GenBank: JQ581351]; PALDA5 [GenBank: JQ581352]; PALDAV23 [GenBank: JQ581350]; Palmascl7 [GenBank: HQ713710]; PANma1 [GenBank: FJ549397]; PLI [GenBank: JX431277]; QROI [GenBank: JX431278]; Roma06 [GenBank: FJ549390]; RP540Cl9 [GenBank: HQ713716]; RPALLcl1 [GenBank: KF220756]; RPALLcl2 [GenBank: KF220757]; RPALLcl3 [GenBank: KF220759]; RPALLcl4 [GenBank: KF220758]; RPALLcl5 [GenBank: KF220755]; SanAntoniocl1 [GenBank: KF220715]; SanAntoniocl2 [GenBank: KF220716]; SanAntoniocl3 [GenBank: KF220717]; SanAntoniocl4 [GenBank: KF220718]; SanAntoniocl5 [GenBank: KF220719]; SC13 [GenBank: AJ130937]; SilvioX10 or X10 cl1 [GenBank: AJ130928]; SJM22cl1 [GenBank: JQ581361]; SJM34 [GenBank: JQ581356]; SJM37 [GenBank: JQ581357]; SJM39cl3 [GenBank: JQ581360]; SJM41 [GenBank: JQ581358]; SJMC12 [GenBank: JQ581359]; SL12 [GenBank: KC951587]; SL7389123 [GenBank: KC951586]; SL890 [GenBank: KC951588]; SLA9cl9 [GenBank: HQ713704]; SLB3cl2 [GenBank: HQ713705]; SLD1Eccl6 [GenBank: HQ713740]; SLD2cl1 [GenBank: HQ713706]; SLDm1cl9 [GenBank: HQ713683]; SLDm2cl10 [GenBank: HQ713684]; SLF5cl10 [GenBank: HQ713708]; SMAcl1 [GenBank: KF220748]; SMAcl3 [GenBank: KF220745]; SMAcl7 [GenBank: KF220746]; SMAcl8 [GenBank: KF220747]; SMAcl9 [GenBank: KF220749]; SN10cl3 [GenBank: HQ713748]; SN5cl7 [GenBank: HQ713736]; SR2cl7 [GenBank: HQ713687]; T09 [GenBank: KC951590]; T23 [GenBank: KC951589]; TCCUSP269 [GenBank: EU856369]; TCCUSP331 [GenBank: EU856370]; TcI [GenBank: KC951574]; Td11cl7 [GenBank: HQ713745]; Td2cl10 [GenBank: HQ713744]; Tdcl9 [GenBank: HQ713743]; TEH or Tehuentepec cl 2 [GenBank: AJ130938]; Til70_Hap7 [GenBank: JF267934]; TmPA1cl6 [GenBank: HQ713741]; TQI [GenBank: JX431279]; TryCC1107 [GenBank: FJ555639]; TryCC1108 [GenBank: FJ549398]; TryCC1109 [GenBank: FJ549399]; TryCC1116 [GenBank: FJ549400]; TryCC1456 [GenBank: FJ555644]; TryCC1620 [GenBank: FJ555646]; TryCC417 [GenBank: FJ002255]; TryCC507 [GenBank: FJ002256]; TryCC540 [GenBank: FJ555634]; TryCC640 [GenBank: FJ549391]; TryCC642 [GenBank: FJ549392]; TryCC876 [GenBank: FJ555636]; TryCC884 [GenBank: FJ555637]; TryCC971 [GenBank: FJ549394]; TVcl9 [GenBank: HQ713742]; U21231 [GenBank: KC951592]; USAARMAcl3 [GenBank: JQ581338]; USAOPOSSUMcl2 [GenBank: JQ581355]; X1082cl9 [GenBank: HQ713702]; X1544cl10 [GenBank: HQ713701]; X236cl8 [GenBank: HQ713703]; X380cl23 [GenBank: HQ713739]; XAL1 [GenBank: JX431280]; XCHcl11 [GenBank: KF220734]; XCHcl12 [GenBank: KF220733]; XCHcl13 [GenBank: KF220732]; XCHcl14 [GenBank: KF220731]; XCHcl15 [GenBank: KF220730]; XE2929 [GenBank: JQ581362]; XE5167cl1 [GenBank: JQ581363]; XE6863/3 [GenBank: FJ549395]; Y721 [GenBank: KC951591]; YAS1cl2 [GenBank: HQ713682]; YB1cl2 [GenBank: HQ713700]; YD1cl5 [GenBank: HQ713699]; YDm1Bcl3 [GenBank: HQ713681]; YDm1Mcl2 [GenBank: HQ713690]; YTT1cl1 [GenBank: HQ713691].

**TcII:** AA12 [GenBank: KC951599]; AA123 [GenBank: KC951601]; AA74 [GenBank: KC951600]; CBB [GenBank: AJ439722, JF267929]; Esmeraldo cl3 [GenBank: AJ130931]; Hap4 [GenBank: JF267931]; Hap5 [GenBank: JF267932]; IB42X [GenBank: FJ183399]; N67213 [GenBank: KC951598]; SL5216 [GenBank: KC951595]; SL53 [GenBank: KC951602]; SL81 [GenBank: KC951594]; TU18 cl2 [GenBank: AJ130932]; U72 [GenBank: KC951593]; Y [GenBank: FJ168768]; Y11 [GenBank: KC951597]; Y77 [GenBank: KC951596].

**TcIII:** AA1323 [GenBank: KC951604]; Arma13 [GenBank: FJ549401]; Arma18 [GenBank: FJ555649]; B13 [GenBank: KC951607]; B432 [GenBank: KC951609]; B9993 [GenBank: KC951608]; CM17 [GenBank: JQ581369]; M5631 [GenBank: AJ439720]; MS2682 or TCCUSP712 [GenBank: EU856374]; MT3663 [GenBank: EU856375]; QJIII [GenBank: FJ549396]; SL423 [GenBank: KC951603]; TryCC1079 [GenBank: FJ555638]; TryCC132 [GenBank: FJ555631]; TryCC1323 [GenBank: FJ555640]; TryCC1356 [GenBank: FJ555641]; TryCC1386 [GenBank: FJ555642]; TryCC1437 [GenBank: FJ555643]; TryCC1457 [GenBank: FJ555645]; TryCC1637 [GenBank: FJ555647]; TryCC844 [GenBank: FJ555635]; TryCC862 [GenBank: FJ183401]; TryCC863 [GenBank: FJ549393]; Y32 [GenBank: KC951605]; Y434 [GenBank: KC951606]; Y489 [GenBank: KC951610].

**TcI**V: 10R26 [GenBank: JQ581366]; 91122102 [GenBank: FJ555648]; 92122102R [GenBank: JQ581365]; 93070103Pcl1 [GenBank: JQ581333]; 9354 [GenBank: JQ581334]; BN34 [GenBank: KC951614]; C732 [GenBank: KC951611]; CACQcl14 [GenBank: HQ713724]; CanIII_cl1 [GenBank: AJ1309292]; CanIIIcl1 [GenBank: JQ581364]; CGcl16 [GenBank: HQ713729]; CP234 [GenBank: KC951613]; CP99 [GenBank: KC951612]; DAcl4 [GenBank: HQ713730]; DogT [GenBank: AJ130930]; DYRcl15 [GenBank: HQ713717]; EEBBcl10 [GenBank: HQ713727]; EHcl1 [GenBank: HQ713728]; EMcl13 [GenBank: HQ713726]; ERAcl2 [GenBank: JQ581367]; FcHcl13 [GenBank: HQ713734]; FECcl15 [GenBank: HQ713722]; Griffin [GenBank: FJ555650]; I88 [GenBank: KC951615]; JEMcl2 [GenBank: HQ713720]; Jose Julio [GenBank: EU856368]; LCVcl3 [GenBank: HQ713718]; LERcl3 [GenBank: HQ713733]; LJVPcl7 [GenBank: HQ713725]; LP12 [GenBank: KC951619]; LP4 [GenBank: KC951618]; M6241 cl6 [GenBank: AJ130933]; MGcl15 [GenBank: HQ713732]; MS2440 [GenBank: EU856371]; NY345 [GenBank: KC951617]; NY4123 [GenBank: KC951616]; Saimiri3cl1 [GenBank: JQ581370]; SEVcl8 [GenBank: HQ713721]; SL87 [GenBank: KC951620]; SMAcl8 [GenBank: HQ713731]; SPcl11 [GenBank: HQ713735]; STC10R [GenBank: FJ555651]; Stc33R [GenBank: AJ439727]; TCCUSP11 [GenBank: EU856376]; TCCUSP1441 [GenBank: EU856380]; TCCUSP337 [GenBank: EU856377]; TCCUSP338 [GenBank: EU856378]; TCCUSP778 [GenBank: EU856379]; TryCC206 [GenBank: FJ555633]; TryCC668 [GenBank: EU856372]; TryCC698 [GenBank: EU856373]; TryCC82 [GenBank: EU856367]; X10610cl5 [GenBank: JQ581368]; Xchcl13 [GenBank: HQ713719]; YLYcl12 [GenBank: HQ713723].

**TcV:** Xd103_Hap8 [GenBank: JF267935]; Xd143_Hap9 [GenBank: JF267936].

**TcVI:** CH2 Hap6 [GenBank: JF267933]; 9280 [GenBank: AJ439725]; CL Brener [GenBank: AJ130935]; Guateque [GenBank: AJ439724]; MN cl2 [GenBank: AJ130934]; SC43 [GenBank: AJ439721]; Tulahuen cl2 [GenBank: AJ130936]; X1092 [GenBank: AJ439726]; X57 [GenBank: AJ439723]; F0V195 [GenBank: EU559322]; MNcl2 [GenBank: EU559325]; TryCC187 [GenBank: FJ555632]; TryCC185 [GenBank: FJ549388]; TryCC186 [GenBank: FJ549389].

***Trypanosoma dionisii*:** TryCC211 [GenBank: FJ900249]; TryCC1110 [GenBank: FJ002263].

***Trypanosoma erneyi*:** TryCC1946 [GenBank: JN040961].
